# Oxidized phagosomal NOX2 complex is replenished from lysosomes

**DOI:** 10.1242/jcs.196931

**Published:** 2017-04-01

**Authors:** Ilse Dingjan, Peter T. A. Linders, Luuk van den Bekerom, Maksim V. Baranov, Partho Halder, Martin ter Beest, Geert van den Bogaart

**Affiliations:** 1 Department of Tumor Immunology, Radboud Institute for Molecular Life Sciences, Radboud University Medical Center, Nijmegen 6525 GA, The Netherlands; 2 Department of Neurobiology, Max-Planck Institute for Biophysical Chemistry, Göttingen 37077, Germany

**Keywords:** NOX2, SNARE, Phagocytosis, Dendritic cell, Reactive oxygen species, Phagosome maturation

## Abstract

In dendritic cells, the NADPH oxidase 2 complex (NOX2) is recruited to the phagosomal membrane during antigen uptake. NOX2 produces reactive oxygen species (ROS) in the lumen of the phagosome that kill ingested pathogens, delay antigen breakdown and alter the peptide repertoire for presentation to T cells. How the integral membrane component of NOX2, cytochrome *b*_558_ (which comprises CYBB and CYBA), traffics to phagosomes is incompletely understood. In this study, we show in dendritic cells derived from human blood-isolated monocytes that cytochrome *b*_558_ is initially recruited to the phagosome from the plasma membrane during phagosome formation. Cytochrome *b*_558_ also traffics from a lysosomal pool to phagosomes and this is required to replenish oxidatively damaged NOX2. We identified syntaxin-7, SNAP23 and VAMP8 as the soluble N-ethylmaleimide-sensitive factor attachment protein receptor (SNARE) proteins mediating this process. Our data describe a key mechanism of how dendritic cells sustain ROS production after antigen uptake that is required to initiate T cell responses.

## Abstract (precis)

**Highlighted Article:** In human dendritic cells, the membrane component of the NADPH oxidase NOX2 complex is initially recruited to phagosomes from the plasma membrane, and oxidized NOX2 complex subunits are replenished from a lysosomal pool.

## INTRODUCTION

Phagocytosis is an essential process by which immune cells clear microbial pathogens as well as apoptotic and necrotic (tumor) cells. Both the plasma membrane and intracellular vesicles contribute to phagosome formation, as intracellular vesicles fuse with the plasma membrane at the cup of the nascent phagosome (Canton and Grinstein, 2014; Fairn and Grinstein, 2012; Lu and Zhou, 2012). The protein and lipid compositions of phagosomes progressively change during phagosome formation and during the subsequent maturation steps, when early phagosomes convert into late phagosomes and then into lysosomes (Canton and Grinstein, 2014; Fairn and Grinstein, 2012; Lu and Zhou, 2012). A key player in the processing of ingested pathogens by phagocytes of the immune system is the NADPH oxidase NOX2 complex. NOX2 consists of the three cytosolic proteins: p47^phox^ (NCF1), p40^phox^ (NCF4) and p67^phox^ (NCF2), and the two integral membrane proteins p22^phox^ (CYBA) and gp91^phox^ (CYBB), which together are called cytochrome *b*_558_ (Bedard and Krause, 2007; Groemping and Rittinger, 2005; Nauseef, 2008). Cytochrome *b*_558_ traffics in complex, and p22^phox^ and gp91^phox^ need to form a stable heterodimer before exit from the endoplasmic reticulum (Casbon et al., 2009; Nauseef, 2008; Zhu et al., 2006). NOX2 produces reactive oxygen species (ROS) that facilitate the killing of the ingested pathogen by neutrophils (Bedard and Krause, 2007; Segal, 2005) and dendritic cells (Vulcano et al., 2004). In dendritic cells, NOX2 also modulates the antigenic repertoire for presentation to both CD4+ and CD8+ T cells (Allan et al., 2014; Bedard and Krause, 2007; Dingjan et al., 2016; Hoffmann et al., 2012; Jancic et al., 2007; Mantegazza et al., 2008; Matsue et al., 2003; Rybicka et al., 2012; Savina et al., 2006; Vulcano et al., 2004). NOX2 localizes to the plasma membrane, to early and recycling endosomes, and to antigen-containing endosomes and phagosomes (Bedard and Krause, 2007; Borregaard et al., 1983; Casbon et al., 2009; Nauseef, 2008). In macrophages and dendritic cells, cytochrome *b*_558_ is recruited to phagosomes from vesicles of a recycling endosomal and lysosomal nature (Casbon et al., 2009; Jancic et al., 2007; Matheoud et al., 2013). How these vesicles are delivered to the phagosome is a field of intense study, and although several players in this process have been identified, such as the small GTPase Rab27a (Jancic et al., 2007) and the Ca^2+^-sensing protein synaptotagmin-11 (Arango Duque et al., 2013), many questions remain. One key open question is which soluble N-ethylmaleimide-sensitive factor attachment protein receptor (SNARE) proteins catalyze the delivery of vesicles containing cytochrome *b*_558_ to phagosomes.

SNARE proteins drive all intracellular membrane fusion reactions in eukaryotic cells (except mitochondrial fusion). Cognate SNAREs present in the donor and acceptor membranes engage and assemble into a four-α-helix coiled-coil bundle. Each helix of this bundle is contributed by a different SNARE motif belonging to separate subfamilies – termed Qa-, Qb-, Qc- and R-SNAREs, respectively (Hong, 2005; Jahn and Scheller, 2006). The SNAREs that catalyze the membrane fusion events underlying phagosome formation and maturation are not well characterized. In the mouse RAW264.7 macrophage cell line, one study has shown a clear switch from syntaxin (stx)12 to stx7 (both Qa) during uptake of sheep red blood cells (Collins et al., 2002). There, stx12 readily disappeared within 10 min after phagosome formation, and this was accompanied by an increase in the levels of stx7. This result suggests a role for stx12 in early, and stx7 in late, phagosomal fusion events, in accordance with their described roles in endosomal maturation (Antonin et al., 2000; Collins et al., 2002; McBride et al., 1999; Mills et al., 2001; Mullock et al., 2000; Nakamura et al., 2000; Prekeris et al., 1998; Pryor et al., 2004; Sun et al., 2003; Ward et al., 2000). For stx7, this agrees well with several quantitative proteomics studies in which stx7 increases in late (>60 min after uptake) compared to early (<30 min) phagosomes for different cell types and phagocytic cargoes (Dill et al., 2015; Goyette et al., 2012; Rogers and Foster, 2007). An exclusive role for stx12 in early phagosomal fusion events is much less clear, as stx12 can be detected on late phagosomes by proteomic analyses (Boulais et al., 2010; Buschow et al., 2012; Jutras et al., 2008; Lee et al., 2010) and western blotting (Fratti et al., 2003; Sakurai et al., 2012). In addition to stx7 and stx12, proteomics studies have identified dozens of other SNARE proteins present on phagosomes (Boulais et al., 2010; Buschow et al., 2012; Dill et al., 2015; Goyette et al., 2012; Jutras et al., 2008; Lee et al., 2010; Rogers and Foster, 2007), but the roles of all these SNAREs remain largely unexplored. The recruitment of some SNAREs, including VAMP8 (R-SNARE) and SNAP23 (both Qb and Qc motifs), can even occur in several phases during phagosome maturation (Rogers and Foster, 2007). All these findings indicate a complex cascade of phagosomal fusion events, with many different SNAREs involved at multiple trafficking steps during phagosomal maturation.

Recently, it has been shown that phagosomal recruitment of cytochrome *b*_558_ is mediated by the R-SNARE VAMP8 (also called endobrevin), and that the intraphagosomal pathogen *Leishmania* suppresses an immune response by selectively cleaving VAMP8 in order to block NOX2 assembly (Matheoud et al., 2013). In this study, we have identified the Q-SNAREs mediating cytochrome *b*_558_ recruitment to phagosomes containing zymosan (particles derived from *Saccharomyces cerevisiae*) in dendritic cells derived from human blood-isolated monocytes. Through a combination of immunofluorescence microscopy, flow cytometry and siRNA knockdown, we demonstrate that gp91^phox^ has already been recruited from the plasma membrane to the phagocytic cup, sometime during formation of the phagosome. Concurrently, gp91^phox^ traffics to phagosomes from an intracellular pool residing in LAMP1-positive compartments. This serves to replenish oxidatively damaged NOX2 at the phagosome. The phagosomal recruitment of gp91^phox^ depends on the SNARE proteins VAMP8, SNAP23 and stx7 that can interact with each other and form a stable complex. siRNA-mediated knockdown of these SNAREs results in reduced recruitment of gp91^phox^ to the phagosome and impairs ROS production. Our findings contribute to our understanding of how dendritic cells are able to sustain the production of ROS for hours after antigen uptake, which is required for antigen processing and presentation (Dingjan et al., 2016; Hoffmann et al., 2012; Jancic et al., 2007; Mantegazza et al., 2008; Matsue et al., 2003; Rybicka et al., 2012; Savina et al., 2006).

## RESULTS

### Gp91^phox^ is recruited to phagosomes from the plasma membrane and from late-endosomes/lysosomes

**Fig. 1.**
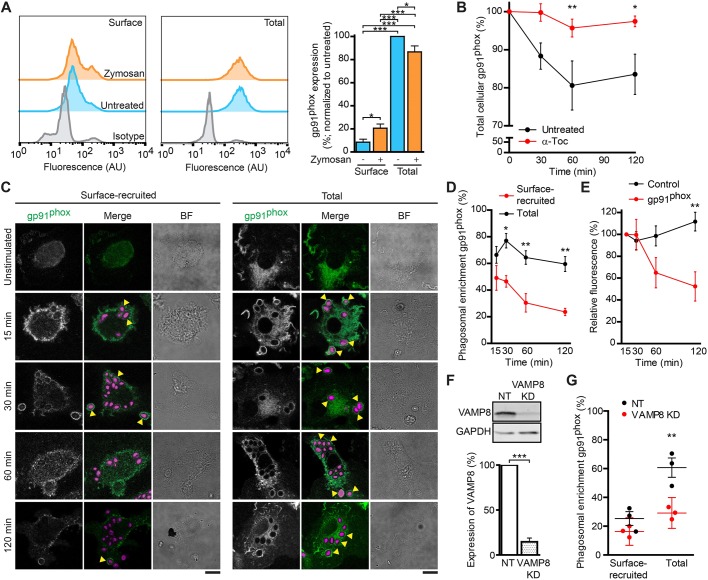
**Recruitment of gp91^phox^ to phagosomes from the plasma membrane and replenishment of oxidized gp91^phox^ from intracellular pools.** (A) Representative flow cytometry histograms (left) and mean fluorescence intensity bar graphs (right) of dendritic cells stained for surface gp91^phox^ (without detergent) or total gp91^phox^ (with detergent) in the presence or absence of zymosan. Gp91^phox^ levels were normalized to those in permeabilized untreated cells (*n*=6 donors; one-way ANOVA with Tukey’s test). (B) Total cellular levels of gp91^phox^ following zymosan stimulation both in the presence and absence of α-tocopherol (α-Toc; *n*=3 donors; two-way ANOVA with Bonferroni's test). (C) Confocal images of dendritic cells that had been pre-incubated with an antibody against gp91^phox^ (recruited to the surface; green in merged image) or with immunostaining against total gp91^phox^ (total) after 15, 30, 60 and 120 min of stimulation with zymosan (magenta). Yellow arrowheads, gp91^phox^-positive phagosomes. BF, brightfield. Scale bars: 10 µm. (D) Quantification of the phagosomal enrichment of gp91^phox^ shown in panel C (*n*=3 donors; two-way ANOVA with Bonferroni's test). (E) Comparison of surface-enriched gp91^phox^ (from panel D) with the presence of a control antibody directed against the zymosan particles (normalized to values at *t*=15 min; *n*=3 donors; two-way ANOVA with Bonferroni's test; representative images are shown in Fig. S1B). (F) Representative western blots and quantification of siRNA-mediated knockdown of VAMP8 relative to that with a non-targeting control siRNA (NT) (*n*=9 donors; two-sided paired Student's *t*-test). KD, knockdown; GAPDH, loading control. (G) Quantification of phagosomal enrichment of surface-recruited and total gp91^phox^ after 60 min of zymosan uptake upon siRNA knockdown of VAMP8 (KD) by immunofluorescence analysis (individual donors shown; two-way ANOVA with Bonferroni's test). Results show means±s.e.m.

In immune cells, gp91^phox^ is present on the plasma membrane and in intracellular compartments (Bedard and Krause, 2007; Borregaard et al., 1983; Casbon et al., 2009; Nauseef, 2008). Activation by immune stimuli can result in enrichment of gp91^phox^ on the plasma membrane (Bedard and Krause, 2007; Borregaard et al., 1983), raising the possibility that gp91^phox^ is recruited to nascent phagosomes from the plasma membrane. In order to determine the source of phagosomal gp91^phox^ in dendritic cells, we first determined which fraction of gp91^phox^ resided on the plasma membrane. Dendritic cells were derived from monocytes from the blood of healthy volunteers and incubated with zymosan particles for 1 h. The presence of gp91^phox^ on the plasma membrane was measured by flow cytometry using an antibody recognizing an extracellular epitope and no permeabilization. This value was compared to that for the total cellular pool of gp91^phox^ with permeabilization (Fig. 1A). Around 10% of the total level of gp91^phox^ was present on the plasma membrane in resting dendritic cells, and this percentage increased to approximately 22% after zymosan stimulation (Fig. 1A). However, we observed a small (∼15%) but significant decrease in total cellular levels of gp91^phox^ upon zymosan stimulation (Fig. 1A,B), suggesting that gp91^phox^ is degraded following zymosan uptake. The decrease of cellular gp91^phox^ could be inhibited by addition of α-tocopherol, a free radical scavenger, during zymosan uptake (Fig. 1B), indicating that the degradation was due to oxidation of gp91^phox^.

We then investigated whether gp91^phox^ is recruited to the phagosome from the plasma membrane during the formation of the phagocytic cup. By pre-incubating the cells with an antibody against gp91^phox^ before zymosan addition, we determined whether plasma membrane gp91^phox^ is taken up together with the zymosan particles during phagocytosis. Most phagosomes were positive for gp91^phox^ that had originated from the plasma membrane at 15 min after uptake, which is the earliest time point at which we obtained a sufficient number of phagosomes for analysis (Fig. 1C, surface recruited). We compared this to the total pool of gp91^phox^ on phagosomes, which we visualized by immunostaining in the presence of detergent (Fig. 1C, total). To quantify the presence of gp91^phox^ on phagosomes, phagosomal membranes were first selected based on morphology (Fig. S1A). Then, we calculated the mean fluorescence intensity of the gp91^phox^ signal at the phagosomal membrane and normalized this by division over the mean fluorescence of the imaged area of the cell to correct for differences in staining efficiencies and varying expression levels among cells and donors. These normalized intensity values allow estimation of phagosomal enrichment for gp91^phox^ in an unbiased manner. Normalized intensity values above 1 indicate enrichment on the phagosomal membrane, whereas values below 1 indicate reduction. The phagosomal enrichment was calculated as the percentage of phagosomes with normalized intensity values above 1. Interestingly, the signal of plasma membrane-originated gp91^phox^ gradually decreased over time after uptake, whereas the signal of total gp91^phox^ on the phagosomes remained stable (Fig. 1D). This decrease of plasma membrane-originated gp91^phox^ signal was caused by sequestration of antibody-labeled gp91^phox^ from the phagosomes and not by degradation or dissociation of the antibody, as experiments with a control antibody (directed against the zymosan particles) showed no decrease in signal (Fig. 1E; Fig. S1B). To test whether gp91^phox^ was replenished at the membrane from intracellular compartments, the same preincubation experiment was performed with dendritic cells transfected with siRNA against VAMP8 (VAMP8 siRNA; 85% knockdown efficiency; Fig. 1F). VAMP8 has been shown to mediate gp91^phox^ recruitment to phagosomes in murine dendritic cells derived from bone marrow (Matheoud et al., 2013). After 60 min of incubation with zymosan, the phagosomal enrichment of plasma membrane-originated gp91^phox^ was equal for cells expressing non-targeting and VAMP8 siRNA (Fig. 1G). However, the total level of gp91^phox^ on phagosomes was only increased in non-targeting siRNA samples and not with VAMP8 siRNA (Fig. 1G). These results not only confirm that VAMP8 mediates the recruitment of gp91^phox^ to phagosomes (Matheoud et al., 2013) but also support the conclusions that gp91^phox^ is initially recruited to phagosomes from the plasma membrane and that there is a subsequent turnover of gp91^phox^ through replenishment from intracellular compartments.

**Fig. 2.**
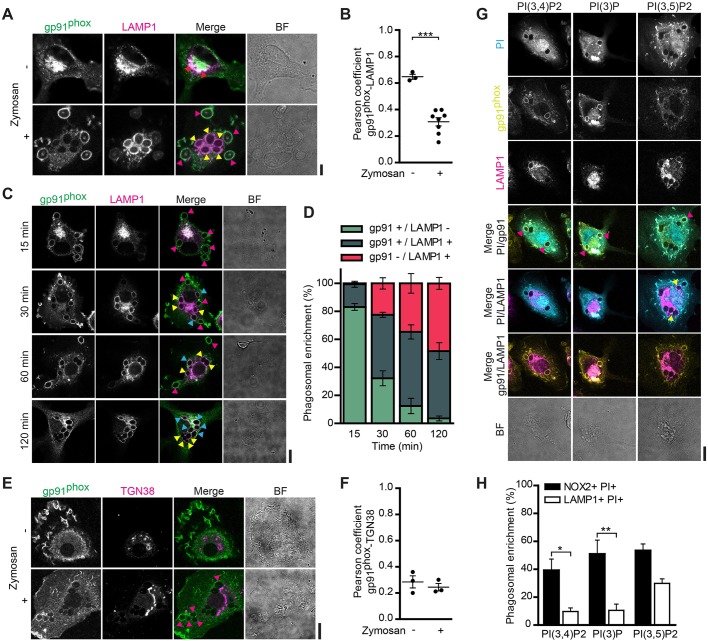
**Gp91^phox^ traffics from LAMP1-rich compartments to phagosomes containing 3-phosphoinositides.** (A) Confocal images of unstimulated and zymosan-pulsed dendritic cells immunostained for gp91^phox^ (green in merge) and LAMP1 (magenta). Red arrowheads, cellular compartments positive for gp91^phox^ and LAMP1; yellow arrowheads, LAMP1-positive phagosomes; pink arrowheads, gp91^phox^-positive phagosomes. BF, brightfield. Scale bar: 5 µm. (B) Pearson correlation coefficients between gp91^phox^ and LAMP1 channels from A (individual donors shown; two-sided unpaired Student's *t*-test). (C) Confocal images of dendritic cells with a pulse of zymosan at 4°C and chased for 15, 30, 60 or 120 min at 37°C that were then immunostained for gp91^phox^ (green in merge) and LAMP1 (magenta). Yellow arrowheads, LAMP1-positive phagosomes; magenta arrowheads, gp91^phox^-positive phagosomes; cyan arrowheads, gp91^phox^ and LAMP1 double-positive phagosomes. Scale bar: 10 µm. (D) Quantification of the phagosomal enrichment of gp91^phox^ and LAMP1 shown in panel C. (E) As panel A but cells were stained for gp91^phox^ (green) and TGN38 (magenta). Scale bar: 10 µm. (F) Pearson correlation coefficients between gp91^phox^ and TGN38 channels from E (individual donors shown). (G) Confocal images of dendritic cells that had been transfected with the GFP-tagged PH domain of TAPP2 [binds to PI(3,4)*P*_2_; cyan in merges], the PX domain of p40^phox^ [PI(3)*P*] or the N-terminus of MCOLN1 [PI(3,5)P_2_] immunostained for gp91^phox^ (yellow) and LAMP1 (magenta). Pink arrowheads, phagosomes positive for both the described phosphoinositide (PI) and gp91^phox^; yellow arrowheads, phagosomes positive for both the described phosphoinositide and LAMP1. Scale bar: 10 µm. (H) Quantification of phagosomal enrichment of gp91^phox^ or LAMP1 on phosphoinositide-positive phagosomes from panel G (*n*=3 donors; one-way ANOVA with Tukey's test). Results show means±s.e.m.

Cytochrome *b*_558_ is known to be recruited to phagosomes from vesicles of an endosomal/lysosomal nature (Arango Duque et al., 2013; Casbon et al., 2009; Jancic et al., 2007; Matheoud et al., 2013), and we tested whether gp91^phox^ was replenished from these compartments in our dendritic cells. Gp91^phox^ clearly overlapped with the late-endosomal/lysosomal marker LAMP1 in unstimulated cells (Fig. 2A), similar to that reported previously for murine dendritic cells and human islet cells (Jancic et al., 2007; Li et al., 2012). However, LAMP1 seemed largely absent from gp91^phox^-positive phagosomes, indicating that LAMP1 and gp91^phox^ localization might be mutually exclusive (Fig. 2A). We calculated Pearson correlation coefficients in order to quantify the overlap of LAMP1 with gp91^phox^ (Fig. 2B). Phagosomes were, again, first selected based on morphology and then the Pearson correlation coefficients of LAMP1 with gp91^phox^ were calculated (Fig. S1A). These were compared with the Pearson correlation coefficients calculated for entire cells in the absence of zymosan. We used Pearson correlation coefficients rather than Mander's overlap coefficients as Pearson correlation coefficients are not influenced by changes in signal intensity (Adler and Parmryd, 2010; Dunn et al., 2011). Interestingly, the Pearson coefficient for LAMP1 with gp91^phox^ dropped from ∼0.7 in cells without zymosan to ∼0.3 with zymosan. To further investigate the distribution of gp91^phox^ and LAMP1 on phagosomes, we performed timecourse pulse–chase experiments. Dendritic cells were pulsed with zymosan at 4°C (at which zymosan will bind to the cells but is not taken up) and subsequently washed and chased at 37°C (a temperature at which phagocytosis occurs). We observed a transition over time with the content of gp91^phox^ on phagosomes gradually decreasing and phagosomal LAMP1 gradually increasing, and this transition occurred via an intermediate phase at which the phagosomes were positive for both proteins (Fig. 2C,D). We could not detect a pool of gp91^phox^ at the Golgi network (Fig. 2E,F), indicating that gp91^phox^ was not recruited to phagosomes from the Golgi. These results indicate that gp91^phox^ traffics from LAMP1-rich late-endosomes/lysosomes to phagosomes containing no or low levels of LAMP1.

For complete assembly and activity of NOX2, not only does cytochrome *b*_558_ need to be present on the phagosome but also the 3-phosphoinositide lipids phosphatidylinositol (3,4)-bisphosphate [PI(3,4)*P*_2_] and/or phosphatidylinositol (3)-phosphate [PI(3)*P*], which interact with p47^phox^ and p40^phox^, respectively, two cytosolic subunits of NOX2 (Anderson et al., 2010; Ellson et al., 2001; Groemping and Rittinger, 2005; Kanai et al., 2001). We tested for the presence of these phosphoinositide lipids on phagosomes by transfecting our dendritic cells with GFP fused to the PH domain of TAPP2 (also known as PLEKHA2) or to the PX domain of p40^phox^, which specifically recognize PI(3,4)*P*_2_ and PI(3)*P*, respectively (Baranov et al., 2016; Kanai et al., 2001; Marshall et al., 2002). We also tested for the presence of late-endosomal/lysosomal phosphatidylinositol (3,5)-bisphosphate [PI(3,5)P_2_] using the N-terminal sequence of MCOLN1 fused to GFP (Baranov et al., 2016; Li et al., 2013). After pulsing the transfected cells with zymosan, we immunolabeled the cells for gp91^phox^ and LAMP1. PI(3,4)*P*_2_ and PI(3)*P* were mostly present on gp91^phox^-enriched phagosomes, but to a lesser extent or not present on LAMP1-enriched phagosomes (Fig. 2G,H). In contrast, PI(3,5)P_2_ was present on phagosomes containing gp91^phox^ or LAMP1. These results show that phagosomes containing gp91^phox^ also contain the 3-phosphoinositides required for NOX2 activity. Taken together, our data show a turnover of NOX2 on phagosomes in which gp91^phox^ is initially recruited from the plasma membrane during formation of the nascent phagosome and, later, oxidatively damaged gp91^phox^ (see Fig. 1B) is replenished from LAMP1-positive compartments of lysosomal nature. Our data further show that LAMP1-enriched phagosomes do not contain high levels of gp91^phox^, nor do they contain sufficient levels of the 3-phosphoinositide species required for NOX2 activity.

### Gp91^phox^ colocalizes with stx7, stx8, SNAP23, Vti1b and VAMP8 on phagosomes

**Fig. 3.**
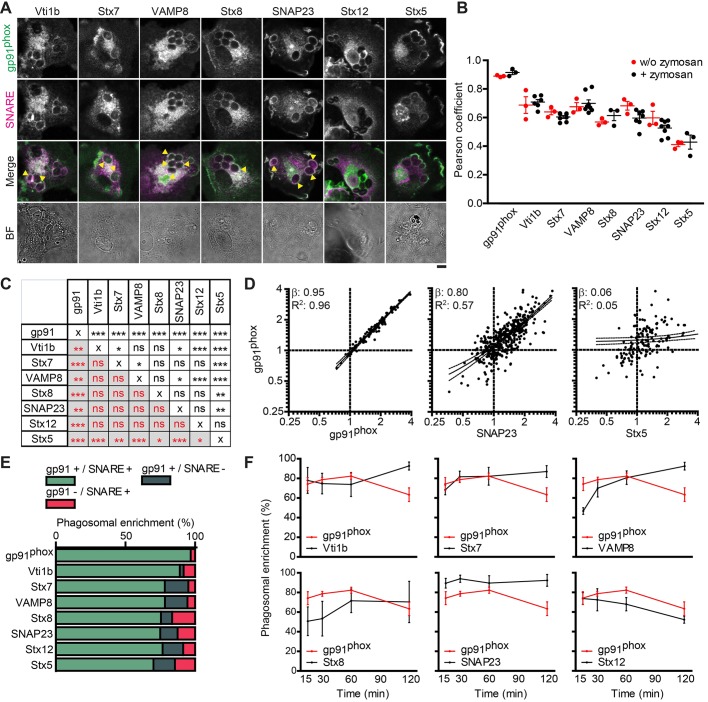
**Gp91^phox^ colocalizes with SNAREs on the phagosomal membrane.** (A) Confocal images of dendritic cells stimulated with zymosan particles for 1 h and immunostained for gp91^phox^ (green in merge) together with Vti1b, stx7, VAMP8, stx8, SNAP23, stx12 or stx5 (magenta). Yellow arrowheads, phagosomes positive for both gp91^phox^ and the indicated SNARE. BF, brightfield. Scale bar: 5 µm. (B) Pearson correlation coefficients for colocalization of gp91^phox^ with the indicated SNAREs for zymosan-containing phagosomes (from panel A) and for unstimulated cells (w/o zymosan; Fig S2A). Colocalization of gp91^phox^ immunostained with both Alexa-Fluor-488 and -568-labeled secondary antibodies is shown as a positive control (individual donors shown). (C) Significance levels (one-way ANOVA with Tukey's test) from panel B. Black text, zymosan-pulsed samples; red text, unstimulated samples. ns, not significant. (D) Normalized staining intensity values of phagosomal gp91^phox^ as a function of normalized intensity values of the indicated SNAREs for individual phagosomes (log2 scale; phagosomes pooled from >3 donors are shown; solid lines, linear regression with 95% confidence intervals; β, regression coefficients; *R*^2^, R-squared values). (E) Quantification of the phagosomal enrichment for gp91^phox^ and/or the indicated SNAREs (from panel D, with normalized intensity values >1). (F) Phagosomal enrichment of gp91^phox^ and the indicated SNAREs following stimulation with zymosan (*n*=6 donors for gp91^phox^; *n*=3 donors for the SNAREs). Results show means±s.e.m.

Next, we addressed the question of which SNAREs mediate the phagosomal recruitment of gp91^phox^ from late-endosomal/lysosomal compartments. We focused on the potential roles of the Q-SNARE proteins SNAP23, Vti1b (Qb), stx7, stx8 (Qc) and stx12 in phagosomal cytochrome *b*_558_ recruitment. We selected these Q-SNAREs for three reasons. First, these SNAREs interact with VAMP8 (Antonin et al., 2000; Murray et al., 2005a,b; Offenhäuser et al., 2011; Sakurai et al., 2012; Wade et al., 2001), which is the R-SNARE involved in phagosomal recruitment of cytochrome *b*_558_ (Matheoud et al., 2013). Second, a recent study with phagosomes purified from human dendritic cells (i.e. the cell type studied here) identified the Q-SNAREs stx7, stx8 and stx12 on phagosomes (Buschow et al., 2012). The presence of SNAP23, stx7, stx12 and Vti1b on phagosomes has been confirmed by immunofluorescence, cellular fractionation and overexpression of tagged fusion proteins (Cai et al., 2011; Cebrian et al., 2011; Collins et al., 2002; Fratti et al., 2003; Nair-Gupta et al., 2014; Sakurai et al., 2012). Third, levels of cytochrome *b*_558_ on phagosomes correlate with phagosomal SNAP23 in a macrophage cell line (Sakurai et al., 2012), and gp91^phox^ trafficking and ROS production could be inhibited by targeting SNAP23 in primary human neutrophils (Uriarte et al., 2011), suggesting a role for this SNARE in the phagosomal recruitment of NOX2 in dendritic cells. We started by investigating the localization of gp91^phox^ relative to that of VAMP8 and to the aforementioned candidate Q-SNARE proteins. As a negative control, we also immunolabeled cells for the ER and Golgi SNARE stx5 (Qa) (Xu et al., 2002; Zhang and Hong, 2001). The colocalization of gp91^phox^ with SNAP23, stx7, VAMP8, Vti1b and stx8 could be clearly observed, both in unstimulated cells and on zymosan-containing phagosomes (Fig. 3A; Fig. S2A). To quantify this colocalization, we performed three unbiased analyses. First, we calculated Pearson correlation coefficients between the channels of interest on the phagosomal membrane (Fig. S1A). These Pearson correlation coefficients were compared with the Pearson correlation coefficients calculated for entire cells in the absence of zymosan. As a positive control for the maximum overlap observable in our samples, we immunolabeled cells with a primary antibody against gp91^phox^ and a combination of secondary antibodies labeled with two different fluorophores. The maximum Pearson coefficient observable in our samples calculated with our positive control was 0.92 (Fig. 3B; Fig. S2B). In contrast to our previous observations with LAMP1 (Fig. 2B), the Pearson correlation coefficients measured for the SNARE proteins with gp91^phox^ were similar between the absence and presence of zymosan (Fig. 3B; Fig. S2B). Pearson coefficients were between 0.5 and 0.7 for gp91^phox^ with Vti1b, stx7, VAMP8, stx8, SNAP23 and stx12 regardless of the presence of zymosan. Colocalization of Stx5 gave a Pearson correlation coefficient below 0.5. The differences in Pearson correlation coefficients for gp91^phox^ with Vti1b, stx7, VAMP8, stx8 and SNAP23 were all found to be statistically significant compared to gp91^phox^ with stx5 (Fig. 3C). Furthermore, the Pearson correlation coefficients for gp91^phox^ with Vti1b and VAMP8 were also significantly higher than the Pearson correlation coefficients for gp91^phox^ with SNAP23, stx7 and stx12. Thus, although we did observe some localization of stx5 at gp91^phox^-positive phagosomes, the localization of Vti1b, stx7, VAMP8, stx8, SNAP23 and stx12 at these phagosomal membranes was more prevalent.

For the second analysis, we correlated the average fluorescence intensity of each of the SNAREs at the phagosomal membranes with that of gp91^phox^ for all individual phagosomes and normalized these by dividing them by the mean fluorescence of the imaged area of the cell (Fig. S1A). We then performed linear regression analysis. Our positive control of gp91^phox^ immunostaining with two differently labeled secondary antibodies showed almost perfect positive correlation (Fig. 3D; β=0.95; *R*^2^=0.96). The correlations of most of the SNAREs with gp91^phox^ were in reasonable agreement with the Pearson correlation coefficients. We observed correlation with the SNAREs Vti1b (β=0.59; *R*^2^=0.39), stx7 (β=0.35; *R*^2^=0.17), VAMP8 (β=0.38; *R*^2^=0.35), stx12 (β=0.46; *R*^2^=0.26) and stx8 (β=0.52; *R*^2^=0.29; Fig. 3D; Fig. S3). No or only weak correlation was observed with stx5 (β=0.06; *R*^2^=0.05). Interestingly, SNAP23 showed the strongest correlation with gp91^phox^ of all SNAREs tested (β=0.80; *R*^2^=0.57), supporting the possibility that cytochrome *b*_558_ delivery to phagosomes is mediated by SNAP23, as suggested previously (Sakurai et al., 2012; Uriarte et al., 2011).

For the third analysis, we calculated the phagosomal enrichment factors (Fig. S1A). Note that this analysis only provides a rough estimate for the enrichment of proteins on phagosomes as the fluorescence on the phagosomal membrane is divided over the average fluorescence over the imaged cell area. We calculated the enrichment factors for phagosomes containing both gp91^phox^ and the SNARE (double positive), and for phagosomes containing only one of these proteins (Fig. 3E). The results of this analysis were in reasonable agreement with those from the Pearson and regression analyses, with the overlap with gp91^phox^ higher for Vti1b, stx7, VAMP8, stx8, SNAP23 and stx12 than for stx5. We also used phagosomal enrichment factors to quantify the kinetics of gp91^phox^ and SNARE recruitment to phagosomes (Fig. 3F). As we expected based on the phagosomal recruitment of gp91^phox^ from the plasma membrane shown by our data (Fig. 1C,D) and on the pulse–chase timecourse experiments with LAMP1 (Fig. 2C,D), gp91^phox^ was already enriched in the majority of the phagosomes within 15 min after stimulation (the earliest time point at which we obtained a sufficient number of phagosomes for analysis). The phagosomal enrichment of gp91^phox^ was stable or increased somewhat up to 60 min after uptake and decreased afterwards. Similarly, SNAP23 and Vti1b remained present at comparable levels over the entire 2 h timecourse of the experiment. The phagosomal enrichment of stx7, VAMP8 and stx8 gradually increased over time, indicating that these SNAREs were recruited from intracellular compartments to the phagosome. In contrast, the phagosomal enrichment of stx12 decreased over time, indicating that it was removed from the phagosomal membrane.

### siRNA knockdown of stx7, SNAP23 and VAMP8 decreases phagosomal NOX2 complex recruitment and activity

**Fig. 4.**
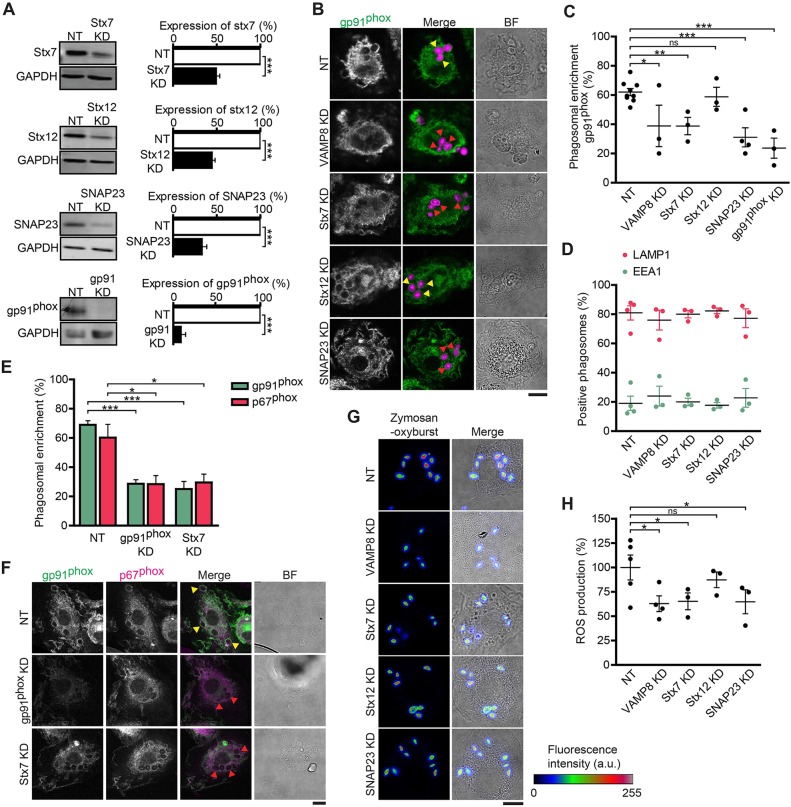
**siRNA knockdown of Stx7, SNAP23 and VAMP8 reduces phagosomal recruitment of cytochrome *b*_558_ and ROS production.** (A) Representative western blots and quantification of siRNA knockdown (KD) of stx7 (51% knockdown efficiency), stx12 (56%), SNAP23 (67%) and gp91^phox^ (92%) relative to levels with a non-targeting siRNA control (NT). GAPDH, loading control (*n*=8 donors; two-sided paired Student's *t*-test). (B) Confocal images of dendritic cells pulsed with Alexa-Fluor-633-conjugated zymosan particles (magenta in merge) and then immunolabeled for gp91^phox^ (green) with knockdown of VAMP8, stx7, stx12 or SNAP23. Yellow arrowheads, phagosomes enriched for gp91^phox^; red arrowheads, phagosomes negative for gp91^phox^. BF, brightfield. Scale bars: 10 µm. (C) Quantification of the phagosomal enrichment of gp91^phox^ from panel B (individual donors shown; one-way ANOVA with Dunnett's test; representative confocal images of gp91^phox^ KD are shown in panel F). ns, not significant. (D) Quantification of the phagosomal enrichment of EEA1 and LAMP1 (individual donors shown; representative confocal images are shown in Fig. S4B). (E) Quantification of the enrichment of gp91^phox^ and p67^phox^ to zymosan-containing phagosomes in dendritic cells with knockdown of gp91^phox^ or stx7 (*n*=3 donors; one-way ANOVA with Tukey's test). (F) Representative confocal images for data presented in E. Yellow arrowheads, phagosomes enriched for gp91^phox^ (green) and p67^phox^ (magenta); red arrowheads, phagosomes negative for gp91^phox^ and p67^phox^. BF, brightfield. Scale bar: 10 µm. (G) Confocal images of zymosan-pulsed dendritic cells with VAMP8, stx7, stx12 or SNAP23 knockdown. Zymosan particles were labeled with ROS-sensitive OxyBURST that becomes fluorescent upon oxidation (shown in rainbow lookup table). Scale bar: 10 µm. (H) Intraphagosomal ROS production calculated from the fluorescence intensities shown in panel G (normalized to those with non-targeting siRNA; one-way ANOVA with Dunnett's test; individual donors shown). Results show mean±s.e.m.

In order to investigate which SNAREs are involved in the phagosomal recruitment of gp91^phox^ from lysosomal compartments, we performed siRNA-mediated silencing of stx7, stx12 and SNAP23 (Fig. 4A; average knockdown efficiencies of 51% for stx7, 56% for stx12 and 67% for SNAP23). As a positive control, we also transfected dendritic cells with siRNA against gp91^phox^ (92% knockdown efficiency; Fig. 4A) and against VAMP8 (85% knockdown efficiency; Fig. 1F), which is the R-SNARE required for phagosomal recruitment of gp91^phox^ (Fig. 1G) (Matheoud et al., 2013). We were unable to obtain substantial knockdown (<25%) of Vti1b and stx8 in dendritic cells. In unstimulated cells, we did not see any obvious rearrangement of gp91^phox^ upon knockdown of the SNAREs, and it still located to the plasma membrane and intracellular pools (Fig. S4A); however, due to the limited resolution of our microscope, we cannot exclude that (part of) the intracellular pool of gp91^phox^ mislocated to other intracellular compartments upon SNARE knockdown. For each condition, we evaluated the enrichment of gp91^phox^ to the phagosome 1 h after uptake of zymosan particles labeled with Alexa-Fluor-633. Each condition was compared to a non-targeting siRNA-transfected condition (Fig. 4B). Compared to results with the non-targeting siRNA, we observed significant loss of phagosomal gp91^phox^ recruitment upon knockdown of VAMP8, stx7 and SNAP23 (Fig. 4C). In contrast, we did not observe loss of gp91^phox^ recruitment upon stx12 knockdown (Fig. 4C). The phagosomal enrichment of LAMP1 and of the early endosomal marker EEA1 were unaffected upon VAMP8, stx7 and SNAP23 knockdown (Fig. 4D; Fig. S4B), indicating that the reduced phagosomal recruitment of gp91^phox^ was not due to a general maturation defect. These results support a role for SNAP23 and stx7 (but not stx12) in the recruitment of gp91^phox^ to phagosomes.

For the complete assembly of NOX2, the cytosolic subunits p47^phox^, p40^phox^ and p67^phox^ need to bind to gp91^phox^ (Bedard and Krause, 2007; Groemping and Rittinger, 2005; Nauseef, 2008). Indeed, recruitment of p67^phox^ to phagosomes was reduced upon knockdown of gp91^phox^ (Fig. 4E,F), confirming that gp91^phox^ is required for NOX2 assembly. In line with this, knockdown of stx7 (i.e. one of the SNAREs required for phagosomal recruitment of gp91^phox^) resulted in decreased recruitment of both gp91^phox^ and p67^phox^ to phagosomes (Fig. 4E,F). To further investigate the potential roles of SNAREs in the assembly of functional NOX2, we measured the effects of SNARE knockdown on phagosomal ROS production. We measured intraphagosomal ROS with the ROS probe OxyBURST Green H_2_DCFDA (2′,7′-dichlorodihydrofluorescein diacetate), which we covalently coupled to zymosan particles. Oxidation of the non-fluorescent H_2_DCF by ROS results in the formation of the highly fluorescent dichlorofluorescein, thereby providing a direct means for detecting ROS in phagosomes of dendritic cells by confocal microscopy (Bass et al., 1983; Hempel et al., 1999) (Fig. 4G). Upon knockdown of VAMP8, stx7 and SNAP23, the OxyBURST fluorescence was decreased compared to that with the non-targeting siRNA (Fig. 4H). We did not observe a significant decrease in ROS production upon stx12 knockdown (Fig. 4H). These findings further support the involvement of stx7, SNAP23 and VAMP8 in gp91^phox^ recruitment to the phagosome. These SNAREs could function in membrane trafficking steps upstream of the recruitment of gp91^phox^ to the phagosome. However, as VAMP8, stx7 and SNAP23 are all located at the phagosomal membrane, it is also conceivable that they directly mediate the delivery of gp91^phox^ to phagosomes, with VAMP8 (R-SNARE) complexing with stx7 (Qa) and SNAP23 (Qb and Qc). A SNARE complex comprising VAMP8, stx7 and SNAP23 has not been described in the literature and we next determined whether these SNAREs can form a complex.

### SNAP23, stx7 and VAMP8 can form a SNARE complex

**Fig. 5.**
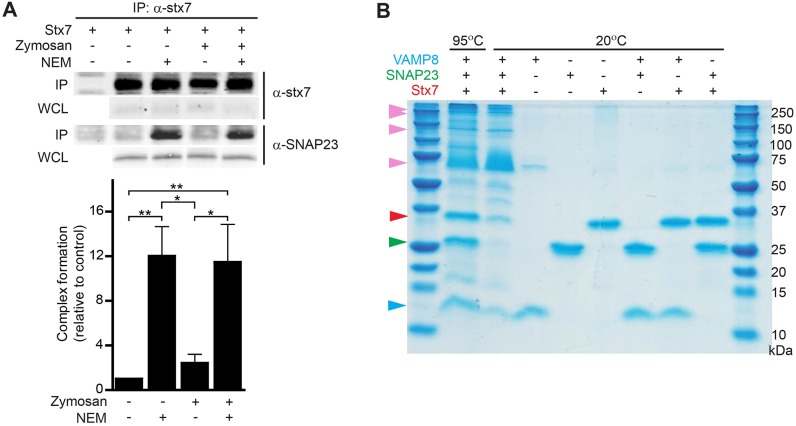
**Complex formation of stx7, SNAP23 and VAMP8.** (A) Immunoprecipitation (IP) of stx7 (α-stx7) from zymosan-pulsed dendritic cells with or without NEM and probed for SNAP23 (α-SNAP23). Shown are a representative western blot and the quantification of the band intensities normalized to control conditions (without zymosan and NEM) (mean±s.e.m. of five donors; one-way ANOVA with Tukey's test). WCL, whole-cell lysate. First lane shows only antibody in buffer without lysate. Results are mean±s.e.m. (B) *In vitro* complex formation of purified full-length VAMP8 (blue arrowhead), SNAP23 (green) and stx7 (red) analyzed by SDS-PAGE and Coomassie staining. Ternary SNARE complexes (multiple bands between ∼250 and 37 kDa; pink arrowheads) are SDS resistant at 20°C but disassemble at 95°C. A representative gel from three experiments is shown.

VAMP8 is well known to complex with stx7 (Antonin et al., 2000, 2002; Pryor et al., 2004; Wade et al., 2001) and with SNAP23 (Paumet et al., 2000; Wang et al., 2004). The interaction of stx7 with SNAP23 has not been described, but stx7 can be immunoprecipitated with overexpressed SNAP23 in J774 macrophages (Sakurai et al., 2012). To investigate whether stx7 complexes with endogenous SNAP23 in dendritic cells, dendritic cell lysates were incubated with antibodies against stx7, and the immunoprecipitates were subjected to western blot analysis for SNAP23 (Fig. 5A). We observed the presence of SNAP23 in the immunoprecipitates, and this presence increased >10-fold when we blocked NSF-mediated SNARE disassembly with N-ethylmaleimide (NEM). The presence of zymosan increased the interaction of SNAP23 with stx7, but not significantly. This experiment demonstrates that SNAP23 interacts with stx7 *in vivo*; we then addressed whether these two SNAREs could form a ternary SNARE complex with VAMP8. We combined recombinantly expressed (in *Escherichia coli*) and purified full-length SNAP23, stx7 and VAMP8 in a stoichiometric ratio. After overnight incubation, this mixture was subjected to SDS-PAGE. A four-helix bundle SNARE complex is very stable and cannot be denatured by SDS at room temperature, but it does disassemble upon heating the sample to 95°C. We observed clear SNARE complex formation (i.e. multiple bands running at high molecular masses) provided that all three SNARE proteins were present, whereas no complexes could be observed with binary mixtures of only two of the SNARE proteins present (Fig. 5B). This experiment demonstrates that SNAP23, VAMP8 and stx7 are capable of forming a SNARE complex *in vitro*.

## DISCUSSION

**Fig. 6.**
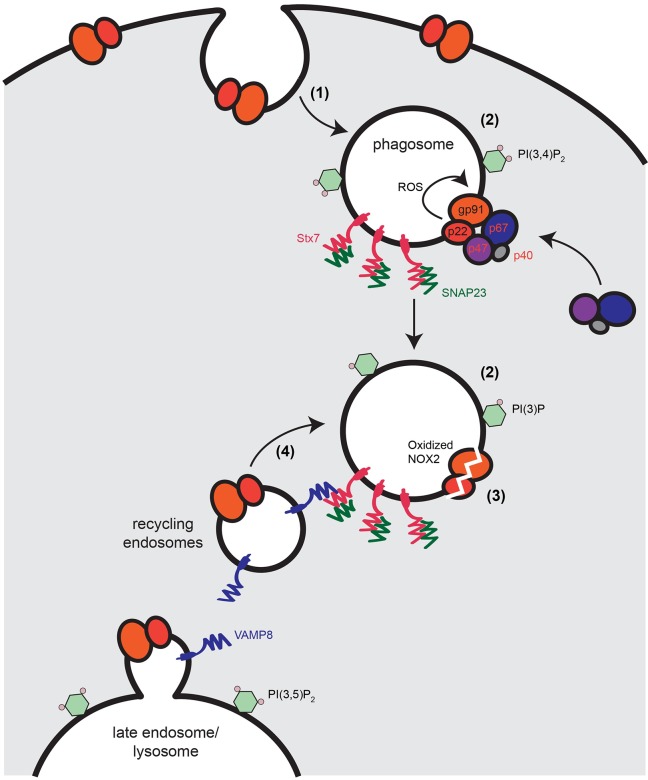
**Model of phagosomal turnover of gp91^phox^.** (1) During phagosome formation, cytochrome *b*_558_ [gp91^phox^ (gp91) and p22^phox^ (P22)] is internalized from the plasma membrane together with zymosan. (2) Cytochrome *b*_558_*-*positive phagosomes contain the 3-phosphoinositides required for NOX2 activity – PI(3,4)*P*_2_ and/or PI(3)*P*. (3) NOX2 produces ROS, which result in auto-oxidation of cytochrome *b*_558_. (4) Phagosomal cytochrome *b*_558_ is replenished from an intracellular pool residing in LAMP1-rich late-endosomes/lysosomes through the action of the SNARE proteins VAMP8, stx7 and SNAP23. p47, p47 ^phox^; p67, p67 ^phox^; p40, p40 ^phox^.

An essential step for the function of NOX2 is the recruitment of its transmembrane component cytochrome *b*_558_ to phagosomes. In this study, we found that gp91^phox^, which traffics together with p22^phox^ (Casbon et al., 2009; Nauseef, 2008; Zhu et al., 2006), is initially internalized together with the zymosan phagocytic cargo from the plasma membrane (Fig. 6). During or after formation of the phagosome, an intracellular pool of gp91^phox^ has already been trafficked to phagosomes from LAMP1-rich compartments of a late-endosomal/lysosomal nature (Fig. 6), as has been reported previously (Jancic et al., 2007; Matheoud et al., 2013). When phagosomes convert into LAMP1-rich lysosomes, they contain no or only low levels of gp91^phox^ and the 3-phosphoinositides PI(3,4)*P*_2_ and PI(3)*P* required for NOX2 activity. Our data show a decrease of cellular levels of gp91^phox^ following zymosan uptake, and this decrease can be blocked by the radical scavenger α-tocopherol; the trafficking from intracellular compartments is likely to serve to replenish gp91^phox^ affected by oxidative damage. Thus, the turnover of the phagosome allows a sustained production of ROS by NOX2 during phagosome maturation. This is likely to be of particular importance for dendritic cell function, where, and in contrast to the more transient oxidative burst in neutrophils and macrophages, sustained ROS production is essential for antigen processing and presentation (Dingjan et al., 2016; Hoffmann et al., 2012; Jancic et al., 2007; Mantegazza et al., 2008; Matsue et al., 2003; Rybicka et al., 2012; Savina et al., 2006).

We identified a role for stx7, SNAP23 and VAMP8 in trafficking of cytochrome *b*_558_ to the antigen-containing phagosome. VAMP8 and SNAP23 have been shown previously to be involved in this process (Matheoud et al., 2013; Sakurai et al., 2012; Uriarte et al., 2011), and our data confirm this. Although SNAP23 has long been considered to be a plasma membrane SNARE (Chamberlain and Gould, 2002; Hong, 2005; Kawanishi et al., 2000; Pagan et al., 2003; Paumet et al., 2000; Tellam et al., 1997; Volchuk et al., 1996; Wang et al., 2004), it is increasingly clear that it also has intracellular functions in many different cell types (Aikawa et al., 2006; Boström et al., 2010; Guo et al., 1998; Martín-Martín et al., 2000; Mollinedo et al., 2006; Nair-Gupta et al., 2014; Sakurai et al., 2012; St-Denis et al., 1999; Takuma et al., 2002; Wang et al., 1997). The localization of SNAP23 to phagosomes was only discovered recently, and it was implied to mediate phagosomal recruitment of gp91^phox^ and MHC class I (Nair-Gupta et al., 2014; Sakurai et al., 2012). Stx7 is widely considered to be a late-endosomal SNARE that mediates fusion of late-endosomes and lysosomes (Antonin et al., 2000; Mullock et al., 2000; Nakamura et al., 2000; Pryor et al., 2004; Ward et al., 2000). Our findings are in line with this given that cytochrome *b*_558_ is recruited from intracellular compartments of a lysosomal nature to phagosomes. As our data show that SNAP23, stx7 and VAMP8 all localize to phagosomes with gp91^phox^ and that they can form a complex together, it seems plausible that these SNAREs directly catalyze the fusion of gp91^phox^-containing vesicles with phagosomes. However, it could also be that these SNAREs catalyze upstream fusion steps needed for cytochrome *b*_558_ trafficking to the phagosome. Accordingly, we cannot exclude the involvement of other SNAREs, including stx8 and Vti1b, due to insufficient knockdown levels. Finally, it may well be that the trafficking of cytochrome *b*_558_ differs among cell types or stimuli, especially considering that zymosan results in much more pronounced NOX2 activity compared to other immune stimuli (Gantner et al., 2003). Stimulus-dependent assembly of NOX2 occurs in primary mouse neutrophils, where NOX2 assembles within seconds on phagosomes bearing serum-opsonized *Staphylococcus aureus*, as opposed to phagosomes containing immunoglobulin G-bound targets, where the NOX2 complex assembles on a tubulovesicular compartment at the base of the emerging phagosome (Anderson et al., 2010).

Possibly, the vesicles trafficking cytochrome *b*_558_ to phagosomes also contain late-endosomal/lysosomal cargo molecules such as the V-ATPase and/or lysosomal cathepsin proteases. This could be the case since the V-ATPase and LAMP1 are recruited to phagosomes in a SNAP23-dependent manner, similar to cytochrome *b*_558_ in a mouse macrophage cell line (Sakurai et al., 2012). In macrophages, V-ATPase subunit a3 is recruited from late-endosomal/lysosomal compartments containing LAMP2 and stx7, and this occurs early during phagosome formation (Sun-Wada et al., 2009), similar to the recruitment of gp91^phox^ observed in our study. However, we found that LAMP1 and gp91^phox^ do not colocalize on phagosomes, and we did not detect a defect of LAMP1 trafficking upon SNARE knockdown. Moreover, both LAMP1 and the V-ATPase are recruited to phagosomes later than gp91^phox^ in human dendritic cells (Mantegazza et al., 2008), and LAMP1 and cathepsins are recruited later than the V-ATPase in murine macrophages (Sun-Wada et al., 2009; Tsang et al., 2000; Yates et al., 2007), making it unlikely that cytochrome *b*_558_-trafficking vesicles also contain these molecules. Another interesting question is how cytochrome *b*_558_ trafficking to the phagosomal membrane is regulated. Key proteins for this process include the small GTPase Rab27a in murine dendritic cells (Jancic et al., 2007) and the Ca^2+^-sensor synaptotagmin-11 in murine macrophages (Arango Duque et al., 2013). The recruitment of gp91^phox^ may also be regulated by phosphorylation of SNAP23, as it has recently been shown that recruitment of MHC class I to phagosomes is promoted by IKK2-dependent phosphorylation of SNAP23 in murine dendritic cells (Nair-Gupta et al., 2014). Similar to NOX2 (Dingjan et al., 2016; Jancic et al., 2007; Mantegazza et al., 2008; Savina et al., 2006), the presence of MHC class I at the antigen-containing phagosome is important for cross-presentation to cytotoxic T lymphocytes by dendritic cells (Burgdorf et al., 2008; Joffre et al., 2012). Our findings contribute to the emerging concept that SNAP23 is a key regulator for dendritic cell function. Targeting SNAP23 and other SNAREs responsible for phagosomal maturation might be a novel strategy to combat autoimmune diseases, infection and cancer.

## MATERIALS AND METHODS

### Cell culture

Primary cultures of human monocyte-derived dendritic cells were generated from peripheral blood monocytes (PBMCs) obtained from buffy coats of healthy individuals (informed consent obtained and approved by RadboudUMC ethical committee) according to institutional guidelines and as described previously (de Vries et al., 2005). Monocytes were differentiated into dendritic cells by culturing for 6 days at 37°C under 5% CO_2_ in complete RPMI-1640 (Gibco, ThermoFisher) supplemented with 10% fetal bovine serum, 2 mM UltraGlutamine (Lonza), 1% antibiotic-antimytotic (AA, Gibco, ThermoFisher), 300 U/ml IL-4 and 450 U/ml granulocyte-macrophage colony-stimulating factor (GM-CSF).

### Antibodies

Primary antibodies used were: mouse-IgG1 anti-gp91^phox^ (D162-3, MBL), rabbit serum anti-SNAP23 (111202, Synaptic Systems), rabbit serum anti-stx8 (110083, Synaptic Systems), rabbit serum anti-stx7 (110072, Synaptic Systems), mouse-IgG1 anti-stx7 (sc-514157, Santa Cruz), rabbit serum anti-stx12 (299022, Synaptic Systems), rabbit serum anti-VAMP8 (104302, Synaptic Systems), rabbit serum anti-Vti1b (164002, Synaptic Systems), rabbit serum anti-stx5 (110053, Synaptic Systems), rabbit serum anti-LAMP1 (L1418, Sigma), rabbit serum anti-TGN38 (sc-27680, Santa Cruz), mouse-IgG1 anti-EEA1 (610456, BD Biosciences), rabbit serum anti-p67phox (07-002, Merck Millipore), rabbit monoclonal-IgG anti-GAPDH (2118, Cell Signaling Technology), mouse-IgG1 anti-FITC (200-602-037, Jackson ImmunoResearch Laboratories) and mouse-IgG1 isotype control (400102, Biolegend) antibodies. The following secondary antibodies were used for immunofluorescence: goat anti-mouse IgG (H+L) Alexa-Fluor-488 or -568-conjugated (A-11029 and A-11031, ThermoFisher) and goat anti-rabbit IgG (H+L) Alexa-Fluor-568 or -647-conjugated (A-11036 and A-21245, ThermoFisher) antibodies. For immunoblotting, we used the secondary antibodies goat anti-rabbit or anti-mouse IgG (H+L) IRDye-800CW-conjugated (926-32211, Li-Cor) antibodies, and for flow cytometry, goat anti-mouse IgG (H+L) Alexa-Fluor-488-conjugated antibodies were used.

### siRNA knockdown assays

Dendritic cells were transfected with siRNA (all ThermoFisher) against gp91^phox^ (5ʹ-CCGAGUCAAUAAUUCUGAUCCUUAU-3ʹ), syntaxin-7 (mix of 3 siRNAs: 5ʹ-GCAGCUGUCAAGGGCAGCAGAUUAU-3ʹ, 5ʹ-GGAGUUGCGAUUAUCAGUCUCAUCA-3ʹ, 5ʹ-GAGAAUCUUCUAUCAGGCAACUUGA-3ʹ), SNAP23 (mix of 3 siRNAs: 5ʹ-GACACCAACAGAGAUCGUAUUGAUA-3ʹ, 5ʹ-GGAUAAUCUGUCAUCAGAAGAAAUU-3ʹ, 5ʹ-CAUAGGCAAUGAGAUUGAUGCUCAA-3ʹ), VAMP8 (mix of 3 siRNAs: 5ʹ-GAGGAAAUGAUCGUGUGCGGAACCU-3ʹ, 5ʹ-GAGGUGGAGGGAGUUAAGAAUAUUA-3ʹ, 5ʹ-CGACAUCGCAGAAGGUGGCUCGAAA-3ʹ) or syntaxin-12 (mix of 3 siRNAs: 5ʹ-GCAACAGUUACAACACUCCACAAAU-3ʹ, 5ʹ-UCACUGAGCAGGAUUUGGAACUUAU-3ʹ, 5ʹ-ACAGUUACAGCGAGCUGCUUACUAU-3ʹ) or with irrelevant ON-TARGET plus Non-Targeting (NT) siRNA#1 (Dharmacon), as described previously (Dingjan et al., 2016).

### Immunoblotting

Knockdown efficiency was analyzed by SDS-PAGE (20 µg/lane; 12% acrylamide). Proteins were transferred to PVDF membranes, blocked with 3% milk and 1% BSA in PBS (137 mM NaCl, 2.7 mM KCl, pH 7.4) and incubated with primary antibodies (1:500 dilution). Protein expression was visualized using IRDye-800- (Li-Cor) labeled secondary antibody (1:5000 dilution). Western blots were imaged using the Odyssey CLx Infrared Imaging System (Li-Cor), and processed with ImageStudio (Li-Cor).

### Microscopy sample preparation

Alexa-Fluor-633-labeled zymosan particles were produced by rehydrating 10 mg zymosan (Z4250, Sigma) in 900 µl of 0.2 M Na_2_CO_3_/NaHCO_3_ pH 9.0. 50 µl of this suspension was subsequently added to a vial containing 220 nmol Alexa-Fluor-633 C5-maleimide (A-20342, ThermoFisher). Unbound dye was removed by vigorous washing with PBS.

For microscope sample preparation, 50,000 dendritic cells were plated on 12-mm-diameter glass coverslips in the presence of unlabeled or Alexa-Fluor-633-labeled zymosan particles in a particle-to-cell ratio of 5:1 in serum-free RPMI medium with 1% AA and 2 mM UltraGlutamine, and incubated at 37°C under 5% CO_2_. For the pulse–chase experiments, cells were incubated for 30 min with zymosan at 4°C, subsequently washed and incubated for 15, 30, 60 or 120 min at 37°C under 5% CO_2_. The cells were washed with PBS and fixed in 4% paraformaldehyde (PFA) in PBS for 15 min at room temperature. Cells were permeabilized with 0.1% (v/v) saponin and blocked with confocal laser scanning microscopy (CLSM) buffer [PBS, 20 mM glycine and 3% (w/v) BSA] for 30 min. For immunostaining, the cells were incubated with primary antibodies (1:200) in CLSM with 0.1% saponin for 1 h at room temperature or overnight at 4°C. Subsequently, cells were washed with PBS and incubated with secondary antibodies (1:400) for 1 h at room temperature. Cells were embedded in 68% glycerol with DAPI.

### Preincubation with antibodies against gp91^phox^

Cells on coverslips were incubated with or without anti-gp91^phox^ antibody (1:400) for 30 min at 4°C. Cells were washed and stimulated with a Alexa-Fluor-633-labeled zymosan particles (5 particles/cell) at 37°C under 5% CO_2_. Subsequently, the cells were fixed, blocked and permeabilized as described above. Samples without anti-gp91^phox^ preincubation were stained with anti-gp91^phox^ antibody (1:200) in CLSM with 0.1% saponin and incubated overnight. This was followed by staining and mounting as described above. For the control antibody experiments, zymosan conjugated to FITC (Baranov et al., 2016) was incubated with Alexa-Fluor-647-labeled anti-FITC antibody (1:200) for 30 min at 4°C, extensively washed and resuspended in RPMI medium. Cells were incubated with these particles and stained with Alexa-Fluor-568-labeled secondary antibody as described above.

### Phosphoinositide transfection

For colocalization experiments with phosphoinositides, cells were transfected with plasmids (10 pg/cell) encoding the PH domain of TAPP2, the PX domain of p40^phox^ or the N-terminus of MCOLN1, as described previously (Baranov et al., 2016). At 7 h post transfection, cells were stimulated with unlabeled zymosan (5 particles/cell) in serum-free RPMI for 1 h at 37°C, and fixed and stained as described before.

### Measurement of phagosomal ROS production

Amine-reactive OxyBURST-Green H_2_DCFDA (3 mg; D-2935, ThermoFisher) was incubated with 10 mg zymosan for 1 h at room temperature. Subsequently, OxyBURST was activated with 1.5 M hydroxylamine. Free dye and hydroxylamine were removed with a Nanosep 300K Omega filter tube (OD300C34, Pall corporation). Cells on coverslips were cultured with OxyBURST-zymosan (4 particles/cell) for 1 h at 37°C under 5% CO_2_, fixed with 4% PFA and mounted as described above.

### Microscopy and colocalization analysis

All samples were imaged with a Leica SP8 confocal laser scanning microscope with a 63×1.20 NA water immersion objective (Leica HC PL APO 63×/1.20 W CORR CS2). All images were evaluated with Fiji (ImageJ 1.49 s). Colocalization and intensity levels were evaluated by first selecting a phagosome based on morphology. The areas corresponding to phagosomal membranes were then selected, and finally colocalization of proteins was calculated using the Pearson correlation coefficient. All images were evaluated independently. At least 25 phagosomes for at least three independent experiments each were analyzed.

### Flow cytometry

Cells were cultured with Alexa-Fluor-633-labeled zymosan (5 particles/cell) with or without 500 µM α-tocopherol in serum-free RPMI with 1% AA and 2 mM UltraGlutamine, and incubated for 30, 60 or 120 min. Cells were then washed with PBS supplemented with 0.5% BSA (in phosphate-buffered albumin PBA) and fixed in 4% PFA in PBS for 4 min on ice. Cells were blocked with PBA supplemented with 2% human serum, or blocked and permeabilized with 0.5% (v/v) saponin in PBA for 10 min on ice. For staining, cells were incubated with anti-gp91^phox^ antibody or isotype control (1:200) in PBA with or without 0.5% saponin for 30 min at 4°C. Subsequently, the cells were washed with PBS and incubated with goat-anti-mouse IgG (H+L) Alexa-Fluor-488 (1:400) in PBA with or without 0.5% saponin for 30 min at 4°C. After incubation, the fluorescence intensity of Alexa-Fluor-488 was measured using flow cytometry (excitation, 488 nm; emission, 530/30 nm; FACSCalibur, BD Biosciences).

### Immunoprecipitation

Cells were incubated with or without zymosan (50 particles/cell) for 45 min at 37°C. Cells were washed twice with PBS and incubated with or without NEM at a final concentration of 2 mM for 15 min at 37°C. NEM was quenched by washing once with 4 mM DTT. Cells were lysed in immunoprecipitation buffer (IP) buffer (20 mM Tris-HCl pH 7.6, 137 mM NaCl, 1% IGEPAL, 2 mM EDTA). Lysates were precleared and incubated with 1 µg of mouse IgG1 anti-stx7 antibody for 30 min on ice, followed by incubation overnight with protein-G–Sepharose at 4°C. Samples were washed with IP buffer, and protein was eluted by boiling in SDS sample buffer. IP samples and 4% of total lysates were analyzed by western blotting for SNAP23 and stx7.

### SNARE complex formation

Fragments of rat full-length VAMP8, rat full-length stx7 and rat full-length SNAP23 were purified as described previously (Antonin et al., 2002; Stein et al., 2009). For assembly of the SNARE complexes, 10 mM of the three protein fragments was incubated overnight at room temperature. Samples were analyzed by SDS-PAGE with colloidal Coomassie Blue (ThermoFisher).

### Statistical analysis

Sample sizes represent the number of individual donors or phagosomes as indicated in the figures. Data were analyzed using one-way ANOVA (following post-hoc Tukey or Dunnett's tests), two-way ANOVA (following post-hoc Bonferroni's test), regression analysis or Student's (un-)paired two-sided *t*-tests. A value of *P*<0.05 was considered statistically significant for all statistical analyses (*P<0.05; **P<0.01; ***P<0.001).

## References

[JCS196931C1] Adler J. and Parmryd I. (2010). Quantifying colocalization by correlation: the Pearson correlation coefficient is superior to the Mander's overlap coefficient. *Cytometry A* 77, 733-742. 10.1002/cyto.a.20896 20653013

[JCS196931C2] Aikawa Y., Lynch K. L., Boswell K. L. and Martin T. F. J. (2006). A second SNARE role for exocytic SNAP25 in endosome fusion. *Mol. Biol. Cell* 17, 2113-2124. 10.1091/mbc.E06-01-0074 16481393 PMC1446080

[JCS196931C3] Allan E. R. O., Tailor P., Balce D. R., Pirzadeh P., McKenna N. T., Renaux B., Warren A. L., Jirik F. R. and Yates R. M. (2014). NADPH oxidase modifies patterns of MHC class II-restricted epitopic repertoires through redox control of antigen processing. *J. Immunol.* 192, 4989-5001. 10.4049/jimmunol.1302896 24778444

[JCS196931C4] Anderson K. E., Chessa T. A. M., Davidson K., Henderson R. B., Walker S., Tolmachova T., Grys K., Rausch O., Seabra M. C., Tybulewicz V. L. J. et al. (2010). PtdIns3P and Rac direct the assembly of the NADPH oxidase on a novel, pre-phagosomal compartment during FcR-mediated phagocytosis in primary mouse neutrophils. *Blood* 116, 4978-4989. 10.1182/blood-2010-03-275602 20813901 PMC3368544

[JCS196931C5] Antonin W., Holroyd C., Fasshauer D., Pabst S., Von Mollard G. F. and Jahn R. (2000). A SNARE complex mediating fusion of late endosomes defines conserved properties of SNARE structure and function. *EMBO J.* 19, 6453-6464. 10.1093/emboj/19.23.6453 11101518 PMC305878

[JCS196931C6] Antonin W., Dulubova I., Arac D., Pabst S., Plitzner J., Rizo J. and Jahn R. (2002). The N-terminal domains of syntaxin 7 and vti1b form three-helix bundles that differ in their ability to regulate SNARE complex assembly. *J. Biol. Chem.* 277, 36449-36456. 10.1074/jbc.M204369200 12114520

[JCS196931C7] Arango Duque G., Fukuda M. and Descoteaux A. (2013). Synaptotagmin XI regulates phagocytosis and cytokine secretion in macrophages. *J. Immunol.* 190, 1737-1745. 10.4049/jimmunol.1202500 23303671

[JCS196931C8] Baranov M. V., Revelo N. H., Dingjan I., Maraspini R., ter Beest M., Honigmann A. and van den Bogaart G. (2016). SWAP70 Organizes the Actin Cytoskeleton and Is Essential for Phagocytosis. *Cell Rep.* 17, 1518-1531. 10.1016/j.celrep.2016.10.021 27806292 PMC5149533

[JCS196931C9] Bass D. A., Parce J. W., Dechatelet L. R., Szejda P., Seeds M. C. and Thomas M. (1983). Flow cytometric studies of oxidative product formation by neutrophils: a graded response to membrane stimulation. *J. Immunol.* 130, 1910-1917. 6833755

[JCS196931C10] Bedard K. and Krause K.-H. (2007). The NOX family of ROS-generating NADPH oxidases: physiology and pathophysiology. *Physiol. Rev.* 87, 245-313. 10.1152/physrev.00044.2005 17237347

[JCS196931C11] Borregaard N., Heiple J. M., Simons E. R. and Clark R. A. (1983). Subcellular localization of the b-cytochrome component of the human neutrophil microbicidal oxidase: translocation during activation. *J. Cell Biol.* 97, 52-61. 10.1083/jcb.97.1.52 6408102 PMC2112494

[JCS196931C12] Boström P., Andersson L., Vind B., Håversen L., Rutberg M., Wickström Y., Larsson E., Jansson P.-A., Svensson M. K., Brånemark R. et al. (2010). The SNARE protein SNAP23 and the SNARE-interacting protein Munc18c in human skeletal muscle are implicated in insulin resistance/type 2 diabetes. *Diabetes* 59, 1870-1878. 10.2337/db09-1503 20460426 PMC2911056

[JCS196931C13] Boulais J., Trost M., Landry C. R., Dieckmann R., Levy E. D., Soldati T., Michnick S. W., Thibault P. and Desjardins M. (2010). Molecular characterization of the evolution of phagosomes. *Mol. Syst. Biol.* 6, 423 10.1038/msb.2010.80 20959821 PMC2990642

[JCS196931C14] Burgdorf S., Schölz C., Kautz A., Tampé R. and Kurts C. (2008). Spatial and mechanistic separation of cross-presentation and endogenous antigen presentation. *Nat. Immunol.* 9, 558-566. 10.1038/ni.1601 18376402

[JCS196931C15] Buschow S. I., Lasonder E., Szklarczyk R., Oud M. M., de Vries I. J. M. and Figdor C. G. (2012). Unraveling the human dendritic cell phagosome proteome by organellar enrichment ranking. *J. Proteomics* 75, 1547-1562. 10.1016/j.jprot.2011.11.024 22146474

[JCS196931C16] Cai D. T., Ho Y. H. S., Chiow K. H., Wee S. H., Han Y., Peh M. T. and Wong S. H. (2011). Aspirin regulates SNARE protein expression and phagocytosis in dendritic cells. *Mol. Membr. Biol.* 28, 90-102. 10.3109/09687688.2010.525756 21231793

[JCS196931C17] Canton J. and Grinstein S. (2014). Priming and activation of NADPH oxidases in plants and animals. *Trends Immunol.* 35, 405-407. 10.1016/j.it.2014.07.007 25127055

[JCS196931C18] Casbon A.-J., Allen L.-A. H., Dunn K. W. and Dinauer M. C. (2009). Macrophage NADPH oxidase flavocytochrome B localizes to the plasma membrane and Rab11-positive recycling endosomes. *J. Immunol.* 182, 2325-2339. 10.4049/jimmunol.0803476 19201887 PMC2666390

[JCS196931C19] Cebrian I., Visentin G., Blanchard N., Jouve M., Bobard A., Moita C., Enninga J., Moita L. F., Amigorena S. and Savina A. (2011). Sec22b regulates phagosomal maturation and antigen crosspresentation by dendritic cells. *Cell* 147, 1355-1368. 10.1016/j.cell.2011.11.021 22153078

[JCS196931C20] Chamberlain L. H. and Gould G. W. (2002). The vesicle- and target-SNARE proteins that mediate Glut4 vesicle fusion are localized in detergent-insoluble lipid rafts present on distinct intracellular membranes. *J. Biol. Chem.* 277, 49750-49754. 10.1074/jbc.M206936200 12376543

[JCS196931C21] Collins R. F., Schreiber A. D., Grinstein S. and Trimble W. S. (2002). Syntaxins 13 and 7 function at distinct steps during phagocytosis. *J. Immunol.* 169, 3250-3256. 10.4049/jimmunol.169.6.3250 12218144

[JCS196931C22] de Vries I. J. M., Adema G. J., Punt C. J. A. and Figdor C. G. (2005). Phenotypical and functional characterization of clinical-grade dendritic cells. *Methods Mol. Med.* 109, 113-126. 15585917 10.1385/1-59259-862-5:113

[JCS196931C23] Dill B. D., Gierlinski M., Härtlova A., Arandilla A. G., Guo M., Clarke R. G. and Trost M. (2015). Quantitative proteome analysis of temporally resolved phagosomes following uptake via key phagocytic receptors. *Mol. Cell. Proteomics* 14, 1334-1349. 10.1074/mcp.M114.044594 25755298 PMC4424403

[JCS196931C24] Dingjan I., Verboogen D. R. J., Paardekooper L. M., Revelo N. H., Sittig S. P., Visser L. J., Mollard G. F. von Henriet S. S., Figdor C. G., ter Beest M. et al. (2016). Lipid peroxidation causes endosomal antigen release for cross-presentation. *Sci. Rep.* 6, 22064 10.1038/srep22064 26907999 PMC4764948

[JCS196931C25] Dunn K. W., Kamocka M. M. and McDonald J. H. (2011). A practical guide to evaluating colocalization in biological microscopy. *Am. J. Physiol. Cell Physiol.* 300, C723-C742. 10.1152/ajpcell.00462.2010 21209361 PMC3074624

[JCS196931C26] Ellson C. D., Gobert-Gosse S., Anderson K. E., Davidson K., Erdjument-Bromage H., Tempst P., Thuring J. W., Cooper M. A., Lim Z. Y., Holmes A. B. et al. (2001). PtdIns(3)P regulates the neutrophil oxidase complex by binding to the PX domain of p40(phox). *Nat. Cell Biol.* 3, 679-682. 10.1038/35083076 11433301

[JCS196931C27] Fairn G. D. and Grinstein S. (2012). How nascent phagosomes mature to become phagolysosomes. *Trends Immunol.* 33, 397-405. 10.1016/j.it.2012.03.003 22560866

[JCS196931C28] Fratti R. A., Chua J., Vergne I. and Deretic V. (2003). Mycobacterium tuberculosis glycosylated phosphatidylinositol causes phagosome maturation arrest. *Proc. Natl. Acad. Sci. USA* 100, 5437-5442. 10.1073/pnas.0737613100 12702770 PMC154363

[JCS196931C29] Gantner B. N., Simmons R. M., Canavera S. J., Akira S. and Underhill D. M. (2003). Collaborative induction of inflammatory responses by dectin-1 and Toll-like receptor 2. *J. Exp. Med.* 197, 1107-1117. 10.1084/jem.20021787 12719479 PMC2193968

[JCS196931C30] Goyette G., Boulais J., Carruthers N. J., Landry C. R., Jutras I., Duclos S., Dermine J.-F., Michnick S. W., LaBoissière S., Lajoie G. et al. (2012). Proteomic characterization of phagosomal membrane microdomains during phagolysosome biogenesis and evolution. *Mol. Cell. Proteomics* 11, 1365-1377. 10.1074/mcp.M112.021048 22915823 PMC3494184

[JCS196931C31] Groemping Y. and Rittinger K. (2005). Activation and assembly of the NADPH oxidase: a structural perspective. *Biochem. J.* 386, 401-416. 10.1042/BJ20041835 15588255 PMC1134858

[JCS196931C32] Guo Z., Turner C. and Castle D. (1998). Relocation of the t-SNARE SNAP-23 from lamellipodia-like cell surface projections regulates compound exocytosis in mast cells. *Cell* 94, 537-548. 10.1016/S0092-8674(00)81594-9 9727496

[JCS196931C33] Hempel S. L., Buettner G. R., O'Malley Y. Q., Wessels D. A. and Flaherty D. M. (1999). Dihydrofluorescein diacetate is superior for detecting intracellular oxidants: comparison with 2′,7′-dichlorodihydrofluorescein diacetate, 5(and 6)-carboxy-2′,7′-dichlorodihydrofluorescein diacetate, and dihydrorhodamine 123. *Free Radic. Biol. Med.* 27, 146-159. 10.1016/S0891-5849(99)00061-1 10443931

[JCS196931C34] Hoffmann E., Kotsias F., Visentin G., Bruhns P., Savina A. and Amigorena S. (2012). Autonomous phagosomal degradation and antigen presentation in dendritic cells. *Proc. Natl. Acad. Sci. USA* 109, 14556-14561. 10.1073/pnas.1203912109 22908282 PMC3437883

[JCS196931C35] Hong W. (2005). SNAREs and traffic. *Biochim. Biophys. Acta* 1744, 120-144. 10.1016/j.bbamcr.2005.03.014 15893389

[JCS196931C36] Jahn R. and Scheller R. H. (2006). SNAREs–engines for membrane fusion. *Nat. Rev. Mol. Cell Biol.* 7, 631-643. 10.1038/nrm2002 16912714

[JCS196931C37] Jancic C., Savina A., Wasmeier C., Tolmachova T., El-Benna J., Dang P. M.-C., Pascolo S., Gougerot-Pocidalo M.-A., Raposo G., Seabra M. C. et al. (2007). Rab27a regulates phagosomal pH and NADPH oxidase recruitment to dendritic cell phagosomes. *Nat. Cell Biol.* 9, 367-378. 10.1038/ncb1552 17351642

[JCS196931C38] Joffre O. P., Segura E., Savina A. and Amigorena S. (2012). Cross-presentation by dendritic cells. *Nat. Rev. Immunol.* 12, 557-569. 10.1038/nri3254 22790179

[JCS196931C39] Jutras I., Houde M., Currier N., Boulais J., Duclos S., LaBoissière S., Bonneil E., Kearney P., Thibault P., Paramithiotis E. et al. (2008). Modulation of the phagosome proteome by interferon-gamma. *Mol. Cell. Proteomics* 7, 697-715. 10.1074/mcp.M700267-MCP200 18156134

[JCS196931C40] Kanai F., Liu H., Field S. J., Akbary H., Matsuo T., Brown G. E., Cantley L. C. and Yaffe M. B. (2001). The PX domains of p47phox and p40phox bind to lipid products of PI(3)K. *Nat. Cell Biol.* 3, 675-678. 10.1038/35083070 11433300

[JCS196931C41] Kawanishi M., Tamori Y., Okazawa H., Araki S., Shinoda H. and Kasuga M. (2000). Role of SNAP23 in insulin-induced translocation of GLUT4 in 3T3-L1 adipocytes. Mediation of complex formation between syntaxin4 and VAMP2. *J. Biol. Chem.* 275, 8240-8247. 10.1074/jbc.275.11.8240 10713150

[JCS196931C42] Lee B.-Y., Jethwaney D., Schilling B., Clemens D. L., Gibson B. W. and Horwitz M. A. (2010). The Mycobacterium bovis bacille Calmette-Guerin phagosome proteome. *Mol. Cell. Proteomics* 9, 32-53. 10.1074/mcp.M900396-MCP200 19815536 PMC2808266

[JCS196931C43] Li N., Li B., Brun T., Deffert-Delbouille C., Mahiout Z., Daali Y., Ma X.-J., Krause K.-H. and Maechler P. (2012). NADPH oxidase NOX2 defines a new antagonistic role for reactive oxygen species and cAMP/PKA in the regulation of insulin secretion. *Diabetes* 61, 2842-2850. 10.2337/db12-0009 22933115 PMC3478534

[JCS196931C44] Li X., Wang X., Zhang X., Zhao M., Tsang W. L., Zhang Y., Yau R. G. W., Weisman L. S. and Xu H. (2013). Genetically encoded fluorescent probe to visualize intracellular phosphatidylinositol 3,5-bisphosphate localization and dynamics. *Proc. Natl. Acad. Sci. USA* 110, 21165-21170. 10.1073/pnas.1311864110 24324172 PMC3876232

[JCS196931C45] Lu N. and Zhou Z. (2012). Membrane trafficking and phagosome maturation during the clearance of apoptotic cells. *Int. Rev. Cell Mol. Biol.* 293, 269-309. 10.1016/B978-0-12-394304-0.00013-0 22251564 PMC3551535

[JCS196931C46] Mantegazza A. R., Savina A., Vermeulen M., Pérez L., Geffner J., Hermine O., Rosenzweig S. D., Faure F. and Amigorena S. (2008). NADPH oxidase controls phagosomal pH and antigen cross-presentation in human dendritic cells. *Blood* 112, 4712-4722. 10.1182/blood-2008-01-134791 18682599 PMC2597138

[JCS196931C47] Marshall A. J., Krahn A. K., Ma K., Duronio V. and Hou S. (2002). TAPP1 and TAPP2 are targets of phosphatidylinositol 3-kinase signaling in B cells: sustained plasma membrane recruitment triggered by the B-cell antigen receptor. *Mol. Cell. Biol.* 22, 5479-5491. 10.1128/MCB.22.15.5479-5491.2002 12101241 PMC133950

[JCS196931C48] Martín-Martín B., Nabokina S. M., Blasi J., Lazo P. a. and Mollinedo F. (2000). Involvement of SNAP-23 and syntaxin 6 in human neutrophil exocytosis. *Blood* 96, 2574-2583. 11001914

[JCS196931C49] Matheoud D., Moradin N., Bellemare-Pelletier A., Shio M. T., Hong W. J., Olivier M., Gagnon E., Desjardins M. and Descoteaux A. (2013). Leishmania evades host immunity by inhibiting antigen cross-presentation through direct cleavage of the SNARE VAMP8. *Cell Host Microbe* 14, 15-25. 10.1016/j.chom.2013.06.003 23870310

[JCS196931C50] Matsue H., Edelbaum D., Shalhevet D., Mizumoto N., Yang C., Mummert M. E., Oeda J., Masayasu H. and Takashima A. (2003). Generation and function of reactive oxygen species in dendritic cells during antigen presentation. *J. Immunol.* 171, 3010-3018. 10.4049/jimmunol.171.6.3010 12960326

[JCS196931C51] McBride H. M., Rybin V., Murphy C., Giner A., Teasdale R. and Zerial M. (1999). Oligomeric complexes link Rab5 effectors with NSF and drive membrane fusion via interactions between EEA1 and syntaxin 13. *Cell* 98, 377-386. 10.1016/S0092-8674(00)81966-2 10458612

[JCS196931C52] Mills I. G., Urbé S. and Clague M. J. (2001). Relationships between EEA1 binding partners and their role in endosome fusion. *J. Cell Sci.* 114, 1959-1965. 11329382 10.1242/jcs.114.10.1959

[JCS196931C53] Mollinedo F., Calafat J., Janssen H., Martín-Martín B., Canchado J., Nabokina S. M. and Gajate C. (2006). Combinatorial SNARE complexes modulate the secretion of cytoplasmic granules in human neutrophils. *J. Immunol.* 177, 2831-2841. 10.4049/jimmunol.177.5.2831 16920918

[JCS196931C54] Mullock B. M., Smith C. W., Ihrke G., Bright N. A., Lindsay M., Parkinson E. J., Brooks D. A., Parton R. G., James D. E., Luzio J. P. et al. (2000). Syntaxin 7 is localized to late endosome compartments, associates with Vamp 8, and Is required for late endosome-lysosome fusion. *Mol. Biol. Cell* 11, 3137-3153. 10.1091/mbc.11.9.3137 10982406 PMC14981

[JCS196931C55] Murray R. Z., Wylie F. G., Khromykh T., Hume D. A. and Stow J. L. (2005a). Syntaxin 6 and Vti1b form a novel SNARE complex, which is up-regulated in activated macrophages to facilitate exocytosis of tumor necrosis Factor-alpha. *J. Biol. Chem.* 280, 10478-10483. 10.1074/jbc.M414420200 15640147

[JCS196931C56] Murray R. Z., Kay J. G., Sangermani D. G. and Stow J. L. (2005b). A role for the phagosome in cytokine secretion. *Science* 310, 1492-1495. 10.1126/science.1120225 16282525

[JCS196931C57] Nair-Gupta P., Baccarini A., Tung N., Seyffer F., Florey O., Huang Y., Banerjee M., Overholtzer M., Roche P. A., Tampé R. et al. (2014). TLR signals induce phagosomal MHC-I delivery from the endosomal recycling compartment to allow cross-presentation. *Cell* 158, 506-521. 10.1016/j.cell.2014.04.054 25083866 PMC4212008

[JCS196931C58] Nakamura N., Yamamoto A., Wada Y. and Futai M. (2000). Syntaxin 7 mediates endocytic trafficking to late endosomes. *J. Biol. Chem.* 275, 6523-6529. 10.1074/jbc.275.9.6523 10692457

[JCS196931C59] Nauseef W. M. (2008). Nox enzymes in immune cells. *Semin. Immunopathol.* 30, 195-208. 10.1007/s00281-008-0117-4 18449540

[JCS196931C60] Offenhäuser C., Lei N., Roy S., Collins B. M., Stow J. L. and Murray R. Z. (2011). Syntaxin 11 binds Vti1b and regulates late endosome to lysosome fusion in macrophages. *Traffic* 12, 762-773. 10.1111/j.1600-0854.2011.01189.x 21388490

[JCS196931C61] Pagan J. K., Wylie F. G., Joseph S., Widberg C., Bryant N. J., James D. E. and Stow J. L. (2003). The t-SNARE syntaxin 4 is regulated during macrophage activation to function in membrane traffic and cytokine secretion. *Curr. Biol.* 13, 156-160. 10.1016/S0960-9822(03)00006-X 12546791

[JCS196931C62] Paumet F., Le Mao J., Martin S., Galli T., David B., Blank U. and Roa M. (2000). Soluble NSF attachment protein receptors (SNAREs) in RBL-2H3 mast cells: functional role of syntaxin 4 in exocytosis and identification of a vesicle-associated membrane protein 8-containing secretory compartment. *J. Immunol.* 164, 5850-5857. 10.4049/jimmunol.164.11.5850 10820264

[JCS196931C63] Prekeris R., Klumperman J., Chen Y. A. and Scheller R. H. (1998). Syntaxin 13 mediates cycling of plasma membrane proteins via tubulovesicular recycling endosomes. *J. Cell Biol.* 143, 957-971. 10.1083/jcb.143.4.957 9817754 PMC2132958

[JCS196931C64] Pryor P. R., Mullock B. M., Bright N. A., Lindsay M. R., Gray S. R., Richardson S. C. W., Stewart A., James D. E., Piper R. C. and Luzio J. P. (2004). Combinatorial SNARE complexes with VAMP7 or VAMP8 define different late endocytic fusion events. *EMBO Rep.* 5, 590-595. 10.1038/sj.embor.7400150 15133481 PMC1299070

[JCS196931C65] Rogers L. D. and Foster L. J. (2007). The dynamic phagosomal proteome and the contribution of the endoplasmic reticulum. *Proc. Natl. Acad. Sci. USA* 104, 18520-18525. 10.1073/pnas.0705801104 18006660 PMC2141809

[JCS196931C66] Rybicka J. M., Balce D. R., Chaudhuri S., Allan E. R. O. and Yates R. M. (2012). Phagosomal proteolysis in dendritic cells is modulated by NADPH oxidase in a pH-independent manner. *EMBO J.* 31, 932-944. 10.1038/emboj.2011.440 22157818 PMC3280544

[JCS196931C67] Sakurai C., Hashimoto H., Nakanishi H., Arai S., Wada Y., Sun-Wada G.-H., Wada I. and Hatsuzawa K. (2012). SNAP-23 regulates phagosome formation and maturation in macrophages. *Mol. Biol. Cell* 23, 4849-4863. 10.1091/mbc.E12-01-0069 23087210 PMC3521691

[JCS196931C68] Savina A., Jancic C., Hugues S., Guermonprez P., Vargas P., Moura I. C., Lennon-Duménil A.-M., Seabra M. C., Raposo G. and Amigorena S. (2006). NOX2 controls phagosomal pH to regulate antigen processing during crosspresentation by dendritic cells. *Cell* 126, 205-218. 10.1016/j.cell.2006.05.035 16839887

[JCS196931C69] Segal A. W. (2005). How neutrophils kill microbes. *Annu. Rev. Immunol.* 23, 197-223. 10.1146/annurev.immunol.23.021704.115653 15771570 PMC2092448

[JCS196931C70] St-Denis J.- F., Cabaniols J.-P., Cushman S. W. and Roche P. A. (1999). SNAP-23 participates in SNARE complex assembly in rat adipose cells. *Biochem. J.* 338, 709-715. 10.1042/bj3380709 10051443 PMC1220107

[JCS196931C71] Stein A., Weber G., Wahl M. C. and Jahn R. (2009). Helical extension of the neuronal SNARE complex into the membrane. *Nature* 460, 525-528. 10.1038/nature08156 19571812 PMC3108252

[JCS196931C72] Sun W., Yan Q., Vida T. A. and Bean A. J. (2003). Hrs regulates early endosome fusion by inhibiting formation of an endosomal SNARE complex. *J. Cell Biol.* 162, 125-137. 10.1083/jcb.200302083 12847087 PMC2172712

[JCS196931C73] Sun-Wada G.-H., Tabata H., Kawamura N., Aoyama M. and Wada Y. (2009). Direct recruitment of H+-ATPase from lysosomes for phagosomal acidification. *J. Cell Sci.* 122, 2504-2513. 10.1242/jcs.050443 19549681

[JCS196931C74] Takuma T., Arakawa T., Okayama M., Mizoguchi I., Tanimura A. and Tajima Y. (2002). Trafficking of green fluorescent protein-tagged SNARE proteins in HSY cells. *J. Biochem.* 132, 729-735. 10.1093/oxfordjournals.jbchem.a003280 12417022

[JCS196931C75] Tellam J. T., Macaulay S. L., McIntosh S., Hewish D. R., Ward C. W. and James D. E. (1997). Characterization of Munc-18c and syntaxin-4 in 3T3-L1 adipocytes. Putative role in insulin-dependent movement of GLUT-4. *J. Biol. Chem.* 272, 6179-6186. 10.1074/jbc.272.10.6179 9045631

[JCS196931C76] Tsang A. W., Oestergaard K., Myers J. T. and Swanson J. A. (2000). Altered membrane trafficking in activated bone marrow-derived macrophages. *J. Leukoc. Biol.* 68, 487-494. 11037969

[JCS196931C77] Uriarte S. M., Rane M. J., Luerman G. C., Barati M. T., Ward R. A., Nauseef W. M. and McLeish K. R. (2011). Granule exocytosis contributes to priming and activation of the human neutrophil respiratory burst. *J. Immunol.* 187, 391-400. 10.4049/jimmunol.1003112 21642540 PMC3582343

[JCS196931C78] Volchuk A., Wang Q., Ewart H. S., Liu Z., He L., Bennett M. K. and Klip A. (1996). Syntaxin 4 in 3T3-L1 adipocytes: regulation by insulin and participation in insulin-dependent glucose transport. *Mol. Biol. Cell* 7, 1075-1082. 10.1091/mbc.7.9.1375 8862521 PMC275959

[JCS196931C79] Vulcano M., Dusi S., Lissandrini D., Badolato R., Mazzi P., Riboldi E., Borroni E., Calleri A., Donini M., Plebani A. et al. (2004). Toll receptor-mediated regulation of NADPH oxidase in human dendritic cells. *J. Immunol.* 173, 5749-5756. 10.4049/jimmunol.173.9.5749 15494527

[JCS196931C80] Wade N., Bryant N. J., Connolly L. M., Simpson R. J., Luzio J. P., Piper R. C. and James D. E. (2001). Syntaxin 7 complexes with mouse Vps10p tail interactor 1b, syntaxin 6, vesicle-associated membrane protein (VAMP)8, and VAMP7 in B16 melanoma cells. *J. Biol. Chem.* 276, 19820-19827. 10.1074/jbc.M010838200 11278762

[JCS196931C81] Wang G., Witkin J. W., Hao G., Bankaitis V. A., Scherer P. E. and Baldini G. (1997). Syndet is a novel SNAP-25 related protein expressed in many tissues. *J. Cell Sci.* 110, 505-513. 9067602 10.1242/jcs.110.4.505

[JCS196931C82] Wang C.-C., Ng C. P., Lu L., Atlashkin V., Zhang W., Seet L.-F. and Hong W. (2004). A role of VAMP8/endobrevin in regulated exocytosis of pancreatic acinar cells. *Dev. Cell* 7, 359-371. 10.1016/j.devcel.2004.08.002 15363411

[JCS196931C83] Ward D. M., Pevsner J., Scullion M. A., Vaughn M. and Kaplan J. (2000). Syntaxin 7 and VAMP-7 are soluble N-ethylmaleimide-sensitive factor attachment protein receptors required for late endosome-lysosome and homotypic lysosome fusion in alveolar macrophages. *Mol. Biol. Cell* 11, 2327-2333. 10.1091/mbc.11.7.2327 10888671 PMC14922

[JCS196931C84] Xu Y., Martin S., James D. E. and Hong W. (2002). GS15 forms a SNARE complex with syntaxin 5, GS28, and Ykt6 and is implicated in traffic in the early cisternae of the Golgi apparatus. *Mol. Biol. Cell* 13, 3493-3507. 10.1091/mbc.E02-01-0004 12388752 PMC129961

[JCS196931C85] Yates R. M., Hermetter A., Taylor G. A. and Russell D. G. (2007). Macrophage activation downregulates the degradative capacity of the phagosome. *Traffic* 8, 241-250. 10.1111/j.1600-0854.2006.00528.x 17319801

[JCS196931C86] Zhang T. and Hong W. (2001). Ykt6 forms a SNARE complex with syntaxin 5, GS28, and Bet1 and participates in a late stage in endoplasmic reticulum-Golgi transport. *J. Biol. Chem.* 276, 27480-27487. 10.1074/jbc.M102786200 11323436

[JCS196931C87] Zhu Y., Marchal C. C., Casbon A.-J., Stull N., von Löhneysen K., Knaus U. G., Jesaitis A. J., McCormick S., Nauseef W. M. and Dinauer M. C. (2006). Deletion mutagenesis of p22phox subunit of flavocytochrome b558: identification of regions critical for gp91phox maturation and NADPH oxidase activity. *J. Biol. Chem.* 281, 30336-30346. 10.1074/jbc.M607191200 16895900

